# Sustained Clinical Improvement in Birch Pollen Allergy After Two Pre‐Seasonal Short Courses of Allergen‐Specific Immunotherapy: A Long‐Term Open‐Label Extension Study

**DOI:** 10.1111/cea.70323

**Published:** 2026-04-30

**Authors:** Ralph Mösges, Friederike Hüffmeier, Ludger Klimek, Oliver Pfaar, Christian Neuhof, Anna Rybachuk, Cengizhan Acikel, Hacer Sahin, Silke Allekotte, Sandra del Pozo, José Luis Subiza, Miguel Casanovas, Mandy Cuevas, Laura Day, Lea Radtke, Esther Raskopf

**Affiliations:** ^1^ ClinCompetence Cologne GmbH Cologne Germany; ^2^ Institute of Medical Statistics and Computational Biology, Faculty of Medicine University of Cologne Cologne Germany; ^3^ Center for Rhinology & Allergology Wiesbaden Germany; ^4^ Department of Otorhinolaryngology Head and Neck Surgery, Section of Rhinology and Allergy, University Hospital Marburg, Philipps‐Universität Marburg Marburg Germany; ^5^ Inmunotek S.L. Madrid Spain; ^6^ Department of Otorhinolaryngology Technische Universität Dresden Dresden Germany

**Keywords:** allergic rhinoconjunctivitis, birch pollen, combined symptom and medication score, mannan conjugate, polymerised allergoid

## Abstract

**Background:**

Mannan‐conjugated allergoids represent an effective option for treating allergic diseases. This study evaluated the clinical impact of the mannan‐conjugated, polymerised birch pollen allergoid EP‐088_T502 in patients with birch pollen‐induced allergic rhinoconjunctivitis over 3 years.

**Methods:**

Following up to a double‐blind, placebo‐controlled dose‐finding study, in this open, long‐term extension study, 154 birch pollen‐allergic patients were enrolled in Germany. Patients were treated with a cumulative dose of 48,000 mTU EP‐088_T502 administered subcutaneously over five pre‐seasonal visits in each of the two treatment years (2021, 2022) with a subsequent follow‐up year in 2023. The primary efficacy endpoint was the combined symptom and medication score (CSMS) during the peak birch pollen season, which was compared with the CSMS of the placebo group during the peak pollen season of 2020. Safety, tolerability, and immunogenicity were also analysed.

**Results:**

Compared to the placebo group of the preceding study, median CSMS during the peak birch pollen seasons showed reductions of 47.5% in 2021 (*p* < 0.001), 51.8% in 2022 (*p* < 0.001), and 36.5% in 2023 (*p* < 0.001). Median daily symptom scores were reduced by 40.7% in 2021 (*p* < 0.001), 40.7% in 2022 (*p* < 0.001), and 25.6% in 2023 (*p* < 0.010). Median daily medication scores reached 0.07 in 2021 and 0.04 in 2022 (*p* < 0.001) and were reduced by 65.9% in 2023 (*p* < 0.003). Immunological responses showed a 4.82‐fold increase in *Bet v1* sIgG4 (*p* = 0.001) and a marked decrease (−65.5%, *p* = 0.001) in the sIgE/sIgG4 ratio after the first treatment phase. EP‐088_T502 demonstrated good safety and tolerability, with only six mild to moderate systemic allergic reactions (Grade I/II). No epinephrine was used.

**Conclusion:**

In this open‐label study, two consecutive years of pre‐seasonal short‐course allergen immunotherapy (AIT) with EP‐088_T502 markedly reduced symptoms and medication need in patients with birch pollen‐induced rhinoconjunctivitis. Persistent therapeutic effects observed during the follow‐up year, although limited by attrition of the study population, suggest sustained clinical improvement and indicate the potential disease‐modifying impact of this treatment regimen.

## Key Messages

- 2 years of SCIT may be sufficient to induce sustained clinical improvements in birch pollen allergic subjects.
- The majority of patients did not require anti‐allergic rescue medication after 2 years of AIT.
- Immunologic parameters confirm desensitisation by mannan‐conjugated allergoids.

## Graphical abstract

3 years follow‐up study to a DBPC dose‐finding trial investigating subcutaneous immunotherapy with T502 in birch‐pollen induced allergic rhinoconjunctivitis. 154 birch‐pollen allergic adults received short‐course treatment of 10,000 mTU/mL of T502 for 2 years and were observed during the next season without treatment. The treatment with mannan‐conjugated birch pollen polymerised allergoid resulted in a sustained reduction of CSMS, also in the group having received only two pre‐seasonal courses of treatment. CSMS, combined symptom and medication score; DBPC, Double‐blind, placebo‐controlled trial; mTU/mL: Mannan therapeutic units per millilitre.

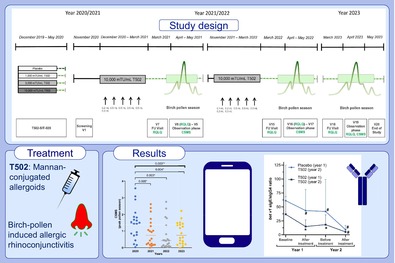

**Abbreviations**
ADRs: allergic adverse drug reactions
AE(s): Adverse Event(s)
AIT: Allergen Immunotherapy
ARC: Allergic Rhinoconjunctivitis
Bregs': regulatory B cells
CSMS: Combined Symptom and Medication Score
DBPC
: Double‐blind, placebo‐controlled trial
DC(s): Dendritic Cell(s)
dMS: daily Medication Score
dSS: daily Symptom Score
DWD: Deutscher Wetterdienst
EAACI: European Academy of Allergy and Clinical Immunology
FDA: Food and Drug Administration
GCP: Good Clinical Practice
IMP: Investigational Medicinal Product
IQR: Interquartile ranges
ITT: Intention to treat
LR: Local Reaction
mTU: Mannan Therapeutic Units
PP: Per Protocol
QoL: Quality of Life
RQLQ: Rhinoconjunctivitis Quality‐of‐Life Questionnaire
S: Safety set
SCIT: Subcutaneous Immunotherapy
SD: Standard deviation
SLIT: Sublingual Immunotherapy
SR: Systemic Reaction
Tregs': regulatory T cells
V: Visit

## 1 Introduction

Allergic rhinoconjunctivitis (ARC) is the most common IgE‐mediated allergic disease, affecting about 400 million people of all ages worldwide. Incidences are increasing further, especially in western industrialised countries [1, 2, 3]. ARC leads to reduced quality of life, can progress to allergic asthma and is associated with a substantial socioeconomic impact [4]. In Northern and Central Europe, the most prevalent and allergenic tree pollen is from birch (Betula), causing mainly intermittent birch‐pollen induced ARC [2, 5]. Due to cross‐reactivity with other pollens, symptoms may persist also outside of the birch pollen season [2]. Therapy is primarily based on symptomatic treatment which is not sufficient in patients with moderate and severe disease [6]. According to guidelines, allergen immunotherapy (AIT) is recommended to reduce symptoms and necessity of antiallergic medication, prevent new sensitisations and progress to allergic asthma [7, 8, 9]. Two established methods exist, the classical subcutaneous immunotherapy (SCIT), which is associated with higher risk of severe allergic adverse drug reactions (ADR), whereas the alternative sublingual route of administration (SLIT) needs higher doses of allergen and seems to be less effective [6, 7, 9, 10]. To date, AIT is the only treatment that enables to achieve tolerogenic response to the causative allergen [8]. Through repeated administration of allergen, regulatory T and B cells (Treg and Breg) increase as well as the production of allergen‐specific IgG antibodies, attenuating IgE activation and the associated allergic reaction [11, 12]. By reprogramming the immune reaction on allergen exposition, AIT leads to long‐sustaining effects even after discontinuation of treatment [13, 14, 15].

Modifying allergen extracts with glutaraldehyde to create polymerised allergoids represents an encouraging approach to enhance the safety of SCIT by reducing allergenicity while maintaining efficacy. The underlying mechanism aims to reduce IgE‐binding epitopes whereas T cell epitopes are preserved [16, 17, 18, 19, 20, 21]. Furthermore, coupling polymerised allergoids to mannan has been shown to enhance the capture of allergens by dendritic cells (DCs) and Treg induction, thereby potentially improving AIT [22, 23]. Initial clinical studies have shown good efficacy and safety of treatment with mannan‐conjugated allergoids [24, 25, 26, 27]. To date, conventional treatment schedules with 3–5 years of treatment and frequent administration of SCIT lead to low adherence and high costs. It is assumed that at least 3 years of AIT are necessary to induce long‐term allergen tolerance [28, 29, 30, 31].

This study aimed at continuing treatment with mannan‐conjugated birch pollen allergoids (EP‐088_T502) in patients who had already participated in a preceding randomised, double‐blind, placebo‐controlled dose‐finding trial T502‐SIT‐020 (EudraCT No.: 2018–002522‐23, 2019/20) [24], for another 2 years (2020/21 and 2021/22), in order to let all patients benefit from the most effective dose resulting from the preceding study. In the preceding trial, patients were treated with different concentrations of the mannan‐conjugated birch pollen allergoids (1000, 3000 or 10,000 mTU/mL) or placebo. The current study was completed by 1 follow‐up year after the discontinuation of treatment. In combination with the dose‐finding trial, it was possible to monitor the treatment over 2 and 3 years and to assess whether 2 years of treatment could be sufficient to induce sustained allergic response improvement in the follow‐up year.

## 2 Methods

### 2.1 Trial Design

**FIGURE 1:**
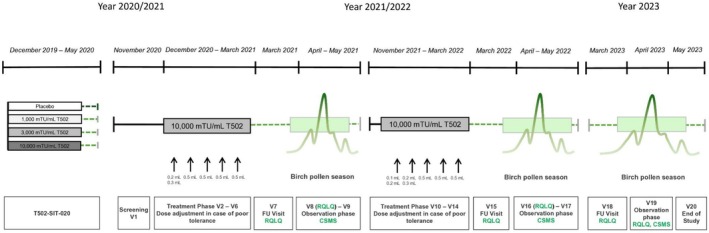
Trial design of the T502‐SIT‐041 trial. Patients received pre‐seasonal subcutaneous injections of EP‐088_T502. Clinical efficacy was assessed by CSMS and RQLQ. mTU/mL, mannan therapeutic units per millilitre; V1‐20, visit 1–20; FU, follow‐up; CSMS, combined symptom and medication score; RQLQ, rhinoconjunctivitis quality‐of‐life questionnaire.

This study was planned as an open‐label, uncontrolled, non‐randomised multicentre follow‐up trial in 15 sites out of 21 centres which contributed to the preceding dose‐finding study in Germany. It involved 20 visits (Figure 1): Study period 2020/2021: 1 screening visit (V1), 5 treatment visits (V2–V6) as pre‐seasonal treatment, 1 pre‐seasonal treatment follow‐up visit (V7), 1 visit at the peak birch pollen season 2021 (V8), and 1 post‐seasonal visit (V9).

Study period 2021/2022: Five treatment visits (V10‐V14) as pre‐seasonal treatment, one pre‐seasonal treatment follow‐up visit (V15), one visit at the peak birch pollen season 2022 (V16), and one post‐seasonal visit (V17).

Observational period 2023: One pre‐seasonal visit (V18), one visit at the peak birch pollen season 2023 (V19), one end‐of‐trial visit after the birch pollen season (V20). The inclusion of 200 patients who had already participated in the preceding dose‐finding trial was planned. Inclusion and non‐inclusion criteria are shown in Data S1.

### 2.2 Ethics Declaration

Relevant trial documentation was submitted to and accepted by the responsible Ethics Committee before the initiation of the study. The trial was approved by the Paul‐Ehrlich‐Institute on 18/DEC/2020 (Reference No.: 4244/01), the regulatory authority in Germany on 01/DEC/2020 (Reference No.: AMG ff‐EK‐416092020) and registered in the EudraCT database (EudraCT No.: 2020–004126‐32). The Declaration of Helsinki (as updated by the World Medical Association in Fortaleza, 2013) [32], the GCP guidelines (CPMP/ICH/135/95), the requirements of national drug and data protection laws as well as other applicable regulatory requirements were strictly followed. All patients gave their written informed consent before entering this trial.

### 2.3 Allergen Immunotherapy (AIT)

AIT was performed with a mannan‐allergoid conjugate of *B. pendula* pollen allergens (EP‐088_T502; 10,000 mTU/mL). Coupling allergens to mannan via glutaraldehyde results in the modification of the native allergen proteins, forming high molecular weight glycoconjugate polymers. Details regarding the manufacturing process are published [22, 23, 33]. The allergenic potency of EP‐088_T502 is expressed in mannan therapeutic units (mTU).

Patients received pre‐seasonally five subcutaneous doses of EP‐088_T502 (10,000 mTU/mL) at five treatment visits in each of the 2 treatment years with intervals of 14 (±6) days (2020/21) or 12–30 days (2021/22). Dose‐splits were conducted at V2, V10, and V11, when doses were split into two injections into 1 arm each (0.2 mL and 0.3 mL at V2 and V11; 0.1 mL and 0.2 mL at V10). At the remaining visits, 1 single subcutaneous injection of 0.5 mL was administered (V3–V6 and V12–V14) (Figure 1). The reduction of the first dose to 0.1 mL and 0.2 mL in the second year of treatment was based on safety and tolerability considerations and was implemented through a substantial modification of the study protocol which was approved by the responsible ethics committee and the Paul‐Ehrlich Institute.

### 2.4 Pollen Measurement Procedures

The entire birch pollen seasons were defined as the period April 1 to April 30 in the years 2021, 2022 and 2023, respectively. The peak birch pollen seasons were the periods of the highest values of the pollen forecast in 14 days in the respective regions according to the German Weather Service (Deutscher Wetterdienst, DWD) and were defined as a constant high pollen concentration of stage 3 (high exposure/> 50 pollen/m^3^) on a scale from 0 to 3.

### 2.5 Endpoints and Statistical Analysis

#### 2.5.1 Assessment of Efficacy—CSMS

To evaluate the clinical impact of EP‐088_T502 treatment in patients with birch pollen‐induced ARC the Combined Symptom and Medication Score (CSMS) during the peak birch pollen season was utilised as primary endpoint. The CSMS is based on the daily symptom score (dSS) and daily medication score (dMS) which are given equal weight with scores ranging from 0 to 3 (dSS: no symptoms–severe symptoms; dMS: no medication–maximal allowed medication) [34]. The symptom score consists of nasal (rhinorrhoea, sneezing, nasal pruritus, nasal congestion) and ocular (ocular pruritus, watery eyes) symptoms. The daily documentation of the scores was carried out via a mobile application (CSMS+/plus Diary App).

Due to the loss of several trial sites (one investigator deceased during the course of the study, others withdrew for administrative reasons), the number of patients was substantially reduced from year 1 to year 4. Consequently, a post hoc analysis was conducted, considering only patients who could be analysed continuously from the dose‐finding trial in 2020 to the follow‐up year in 2023. The complete results of this analysis are presented in Table S4.

#### 2.5.2 Assessment of Efficacy—Clinical Endpoints

Secondary efficacy endpoints included health related quality of life (QoL) which was assessed before and during the birch pollen seasons (V7, V8, V15, V16, V18 and V19). Therefore, the Rhinoconjunctivitis Quality‐of‐Life Questionnaire (RQLQ) according to Juniper & Guyatt [35] in its validated German form [36, 37] was used. The RQLQ was designed as a disease‐specific questionnaire that measures health related QoL in patients with rhinitis and rhinoconjunctivitis, where lower RQLQ scores indicate better QoL [35].

Clinical immunogenicity investigations were based on serum levels of *Bet v1* specific IgE, IgG, and IgG4 at V1, V7 or early termination follow‐up visit. The responsible laboratories for component‐resolved birch pollen‐specific IgE, IgG and IgG4 measurements assessed by ImmunoCAP Method (Thermo Fisher) were MLM Medical Labs (Mönchengladbach, Germany) for IgE and MVZ Stein (Mönchengladbach, Germany) for IgG and IgG4.

#### 2.5.3 Safety and Tolerability

Solicited local adverse events (AEs), namely wheals and redness at the injection site, were measured by investigators and reported 30 min after each injection. Additionally, patients documented local reactions (LRs) and other AEs (e.g., systemic reactions (SRs)) in the evening of the injection and during 2 subsequent days using diary cards. Unsolicited AEs included all local symptoms other than wheals or redness and all SRs. AEs were coded using MedDRA, Version 26.0.

In this trial, Fexofenadine tablets (180 mg) served as on demand treatment of local side effects induced by the IMP (in accordance with the Summary of Product Characteristics). Patients were provided with 10 tablets before the first administration and the use of rescue medication was recorded in the diary cards.

Physical examinations and vital signs were assessed at all visits. For the evaluation of safety laboratory values (blood count, renal and liver function‐related parameters) blood samples were collected at V1 and V7 or early termination follow‐up visit.

### 2.6 Statistical Analysis

Statistical analysis was performed using SPSS Statistics for Windows, Version 27.0 and 29.0 (Armonk, NY: IBM Corp.) according to a predefined statistical analysis plan.

The Safety set (S set) included all patients who have been exposed to the IMP at least once. The modified ITT (mITT) set, being the population of the primary analysis, comprised all patients with at least one entry in the CSMS+/plus diary app. Immunogenicity was analysed in the Immunogenicity set, including all patients with at least two measurements of at least one immunogenicity parameter in the first treatment year. Safety and clinical tolerability were analysed in the S set.

Statistical evaluations for primary and secondary endpoints were performed using descriptive statistics. The course of CSMS and RQLQ over 3 years was also analysed exploratively. No hypotheses were tested.

Comparisons were performed between the years as well as between the placebo group from the preceding study and the patients who had been continuously treated with the highest dose of 10,000 mTU/mL.

Distribution of continuous variables were analysed using Shapiro–Wilk test and also plots and graphs were used to check the normality of data. Data was presented in median and interquartile ranges (IQR) and/or means ± standard deviation (SD). All the exploratory significance tests were two‐sided. and *p*‐values are given without α‐adjustment for multiple testing. Comparison of categorical variables between groups was performed by Chi square or Fisher's exact test. Comparison between two different time points was performed by paired sample *t‐*test or Wilcoxon test. Average CSMS, dMS, and dSS values were calculated for each day per patient over the entire and peak pollen seasons 2021, 2022, and 2023. Descriptive statistical analysis of CSMS, dMS, and dSS was conducted on the basis of those values. The post hoc analysis of the original placebo group was conducted using the Friedman test (repeated measures), applying Nemenyi's procedure for multiple pairwise comparisons.

Immunogenicity parameters for each time point were analysed descriptively. Serum levels, the ratios IgE/IgG4, and their absolute and relative differences were calculated.

GraphPad Prism version 10 for Windows (GraphPad Software, San Diego, California USA) was used for the creation of graphs.

## 3 Results

### 3.1 General Trial Data and Baseline Characteristics

**FIGURE 2:**
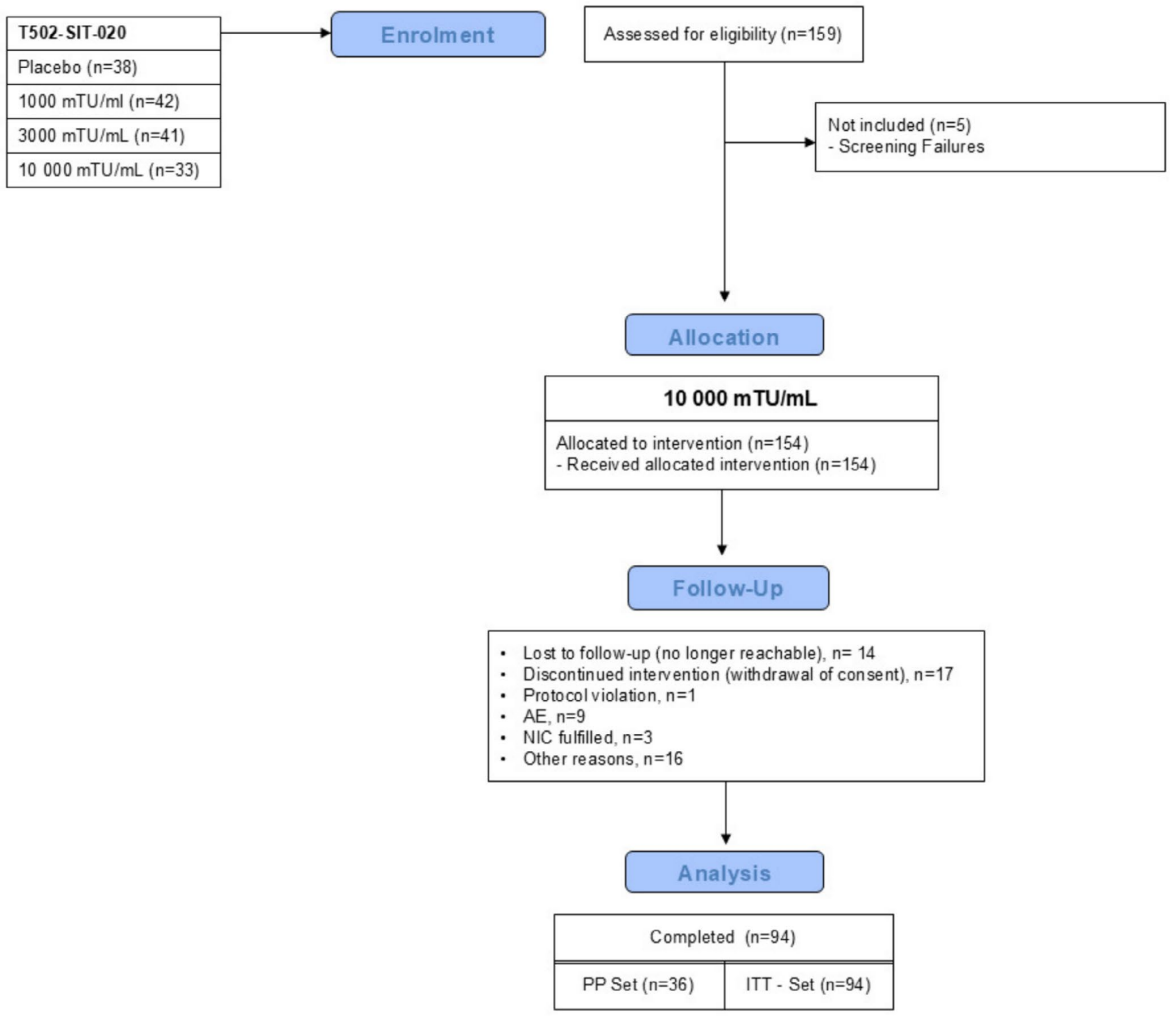
CONSORT flow chart of the trial. ITT, intention to treat; NIC, Non‐Inclusion criteria; PP, per protocol.

The current trial was conducted between December 2020 (first screening visit) and June 2023 (last end‐of‐trial visit). Overall, the mean/median study duration was 833/861 days. Over the 2 treatment phases, the mean/median treatment duration was 94/107 days. One hundred and fifty‐four patients of the 159 screened patients were allocated to the intervention. Of these, 143 patients completed year 1, 116 patients completed year 2 and 94 patients completed the study in 2023. Of the 60 patients in the placebo group of the preceding study, 38 participated in the T502‐SIT‐041 study (Figure 2). Demographics, baseline characteristics and asthma status are shown in Table 1.

**TABLE 1:** Demographics and baseline characteristics of the safety set.

Characteristics	Treatment groups				
	Placebo	1000	3000	10,000	Total
Patients (*N*)	38	42	41	33	154
Female (*N*)	18	19	28	14	79
Female (%)	47.4%	45.2%	68.3%	42.4%	51.3%
Male (*N*)	20	23	13	19	75
Male (%)	52.6%	54.8%	31.7%	57.6%	48.7%
Age mean ± SD (years)	37.45 ± 10.06	42.1 ± 12.04	39.54 ± 11.67	40.97 ± 11.51	40.03 ± 11.39
Height mean ± SD (cm)	173 ± 9	174 ± 10	171 ± 10	175 ± 12	173 ± 10
Body weight mean ± SD (kg)	79 ± 19	83 ± 16	75 ± 17	80 ± 15	79 ± 17
Asthma (*N*)	12	12	11	14	49
Asthma (%)	31.6%	28.6%	26.8%	42.4%	31.8%
Allergic Asthma (*N*)	11	12	10	14	47
Allergic Asthma (%)	28.9%	28.6%	24.4%	42.4%	30.5%

### 3.2 CSMS During the Peak Birch Pollen Season (mITT Set)

**FIGURE 3:**
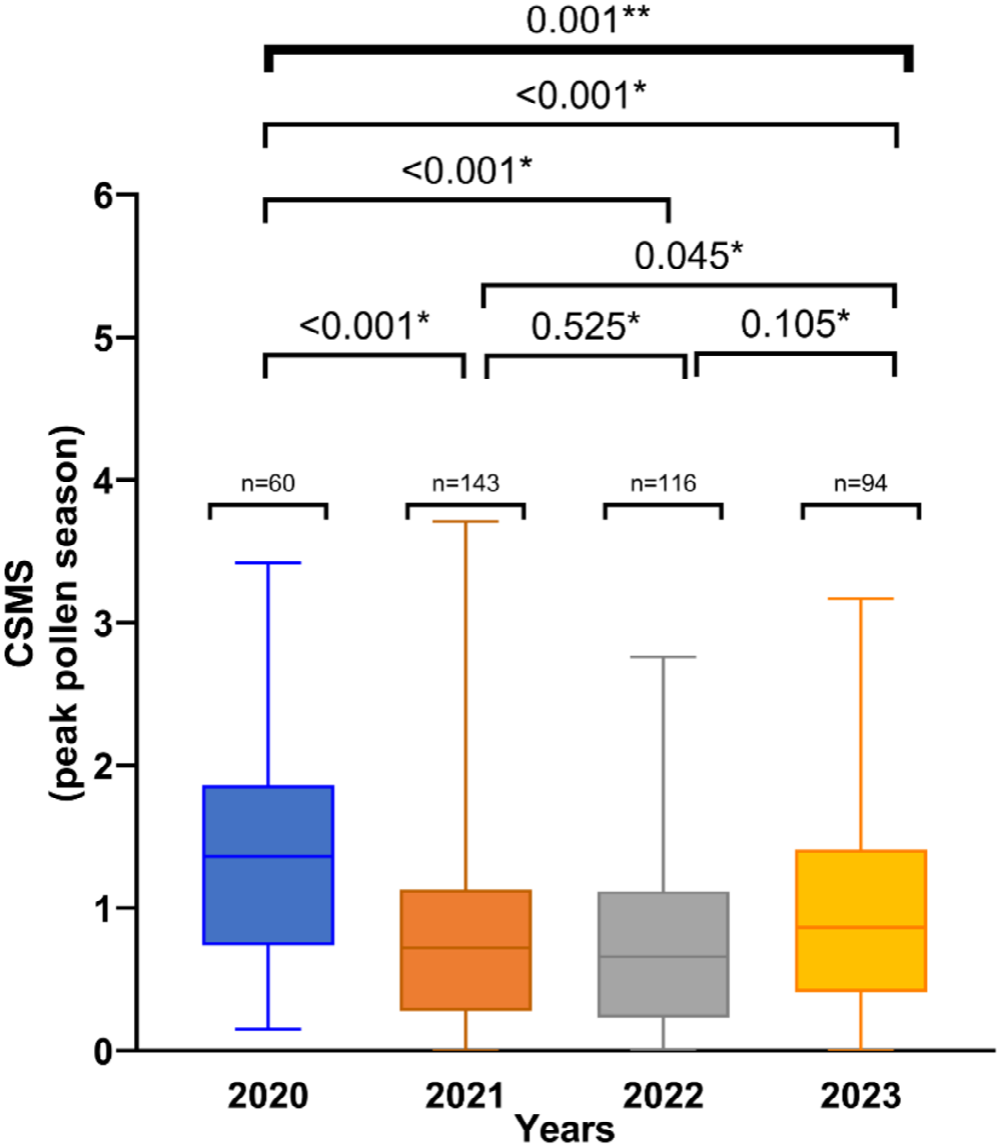
CSMS during the peak birch pollen seasons 2020–2023. Comparison of all participants in the T502‐SIT‐041 study (2021–2023) with placebo participants from the T502‐SIT‐020 study (2020). Data is presented as box plots including minimum, maximum, median, 25th and 75th percentile. *Mann Whitney U tests were used for pairwise comparisons providing the unadjusted *p* values for each comparison. **Kruskal Wallis test was employed to identify differences across multiple comparisons indicating *p* value.

In comparison to the baseline value (placebo group (*N* = 60) in the preceding trial (EudraCT No.:2018–002522‐23), collected during the peak birch pollen season 2020), treatment with EP‐088_T502 reduced the CSMS by 47.5% (median score: 1.37 vs. 0.72 *p* < 0.001) during the peak birch pollen season 2021 (Figure 3). This difference is clinically relevant. The CSMS further decreased during the peak birch pollen season 2022 by a total of 51.8% when compared to 2020 (*p* < 0.001) to 0.66. In comparison to 2020, the CSMS was reduced by 36.5% to 0.87 in the follow‐up year 2023 (*p* < 0.001).

Regarding the dSS alone, treatment with EP‐088_T502 reduced symptoms in 2021 by 40.7% (median score: 0.51) compared to placebo in 2020 (median score: 0.86; *p* < 0.001). In the 2022 birch pollen season, the dSS was again 0.51 (−40.7% in comparison to 2020, *p* < 0.001). In the follow‐up year the dSS was 0.64 (−25.6% when compared to 2020, *p* < 0.010). The median dMS in 2020 was 0.44. In 2021 and 2022 the dMS decreased to a median score of 0.00 (*p* < 0.001). In 2023 the dMS was 0.15 (−65.9% when compared to 2020, *p* < 0.003). Comparing patients treated with placebo in the dose‐finding trial to those who continuously received treatment with the highest dose (10,000 mTU/mL) across both trials, median CSMS values after the first year of treatment (2021) were still 57.45% higher in the original placebo group (0.74 vs. 0.47, *p* = 0.406). In the follow‐up period, median CSMS values showed convergence (0.75 vs. 0.79, *p* = 0.537) (Table S4).

**FIGURE 4:**
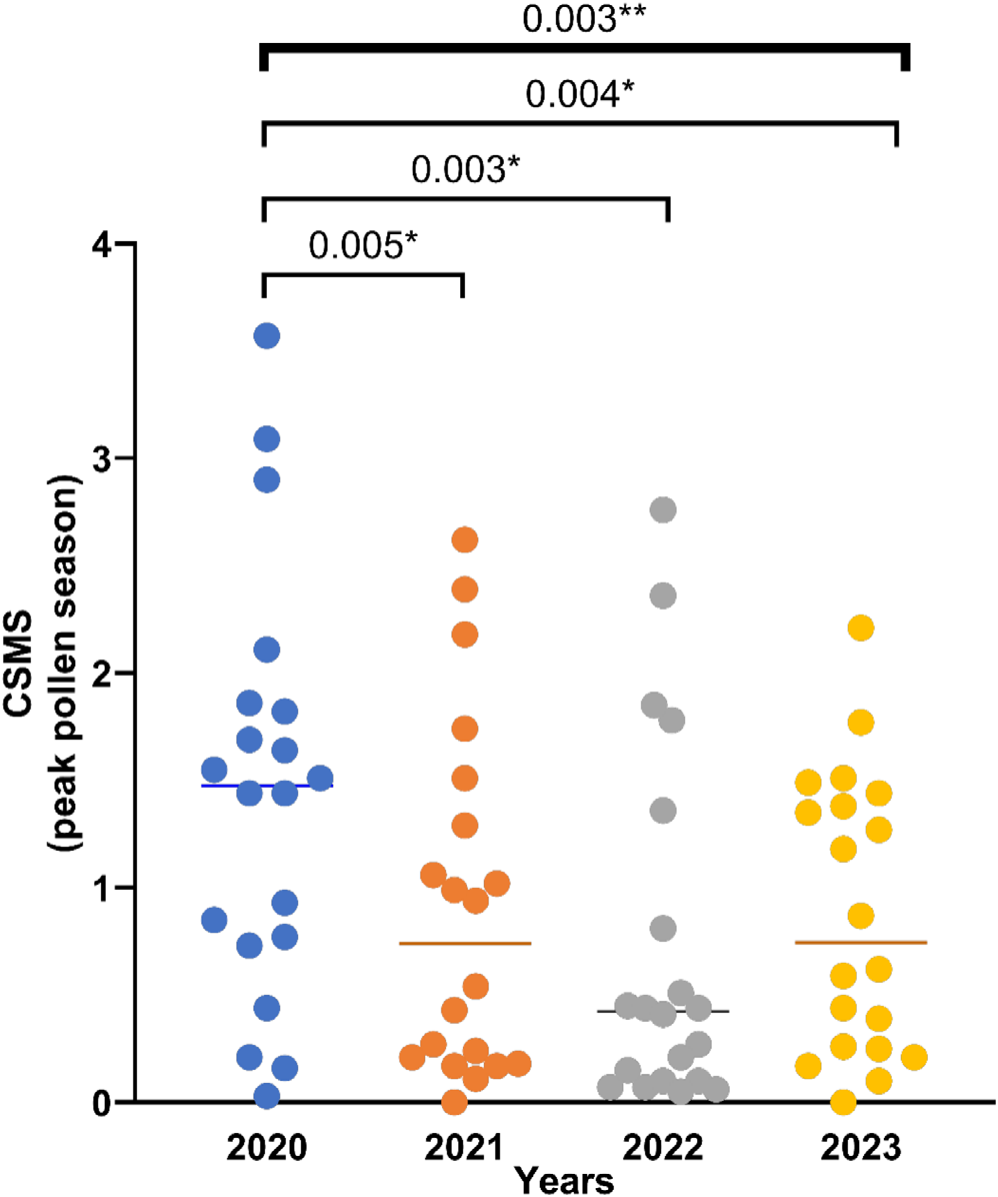
CSMS during the peak birch pollen season. Post hoc analysis of 20 patients originating from the placebo group in the preceding study who completed the entire study in 2023 and received 5 injections of 0.5 mL of EP‐088_T502 in the years 2021 and 2022. Data is presented as scatter dot plots including median value. *Wilcoxon test was used for pairwise comparisons providing the unadjusted *p* values for each comparison. **Friedman test was employed to identify differences across multiple comparisons indicating significant *p* value.

Similar results could be demonstrated for the entire pollen season. In a post hoc analysis of patients originating from the placebo group in the preceding study who completed the entire study in 2023 and received all doses of EP‐088_T502 (*N* = 20), assessment of the CSMS showed improved values still in the follow‐up year (Figure 4). In 2021, the median CSMS was reduced by 50% compared to the baseline from the preceding study in 2020 (0.74 vs. 1.48, *p* = 0.005). The CSMS was reduced further in 2022 to 0.43, representing a 70.95% reduction (*p* = 0.003) in comparison to 2020. In the follow‐up year, CSMS values increased (0.75) but were still 49.33% lower (*p* = 0.004) compared to baseline values in 2020. Additionally, a Friedman test was conducted within this group across the different years, revealing no relevant difference between 2022 and 2023 (*p* = 0.998), which suggests a sustained clinical benefit following treatment discontinuation (Figure 4). The comparison between 2020 and 2023 revealed a difference (*p* = 0.004, Wilcoxon test), with a moderate‐to‐large effect size (*r* = 0.644) [38].

Considering only the group of participants who completed this trial (*N* = 94) 1 year treatment with EP‐088_T502 showed a CSMS reduction of 60.1% (median score: 1.48 vs. 0.59, *p* = 0.003) compared to the CSMS results of those 20 patients within this group who received placebo in 2020 (Figure S2 and Table S5). The CSMS also decreased in 2022 (by a total of 57.5% when compared to 2020, *p* = 0.003) to 0.63. In comparison to 2020, the CSMS was reduced by 41.2% to 0.87 in the follow‐up year 2023 (*p* = 0.025).

### 3.3 Rhinoconjunctivitis Quality‐of‐Life Questionnaire (RQLQ)

QoL improved under treatment with EP‐088_T502, as reflected by the reduction in median RQLQ scores. Although the median RQLQ at the peak of the 2021 birch pollen season (0.82) was higher than the pre‐season value (0.54; +51.9%, n.s.), it represented a notable improvement (49.1% reduction) compared to the peak of the 2020 season (1.61). Similarly, during the 2022 peak pollen season, the RQLQ increased from the pre‐season level (0.59) to 0.88 (+49.1%), but remained substantially improved (45.3% lower) compared to 2020. In 2023, the peak season RQLQ score was 1.29, still indicating a meaningful improvement (19.9% reduction) compared to the baseline year of 2020 (Supporting Information D, Figure S3).

### 3.4 Development of Immunological Parameters (Immunogenicity Set)

**FIGURE 5:**
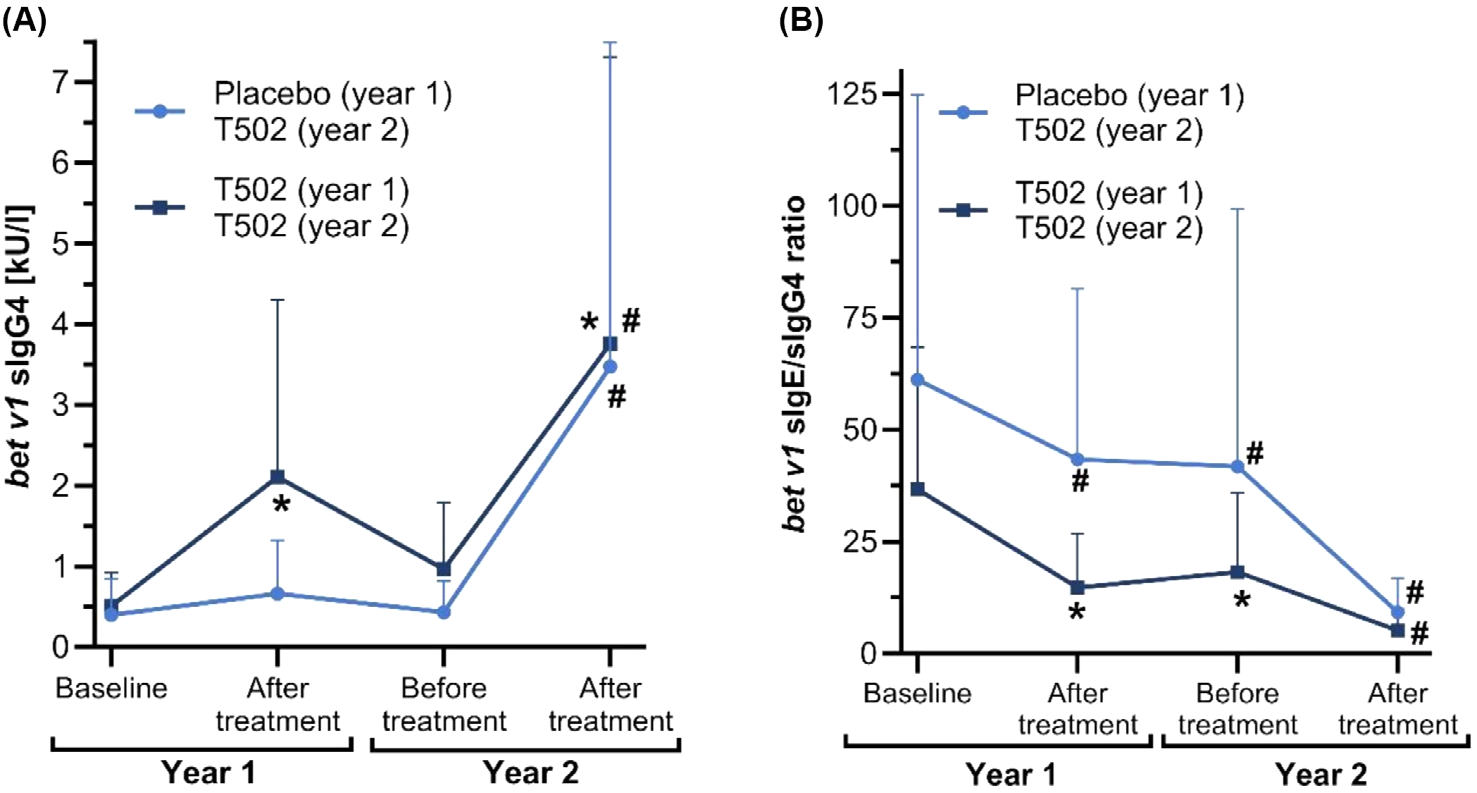
(A) *Bet v1* sIgG4 levels (kU/L) and (B) *Bet v1* sIgE/sIgG4 ratio at baseline (V1) and after treatment (V7). Data is expressed as mean + SD. *p < 0.01 in comparison to baseline, #*p* < 0.005 in comparison to placebo.

From V1 to V7, a 4.82‐fold increase of *Bet v1* sIgG4 could be shown in the Immunogenicity set (*p* = 0.001; Figure 5A). Likewise, *Bet v1* sIgG levels increased 2.73‐fold under treatment with EP‐088_T502 from V1 to V7 (*p* = 0.001).

Values of Bet v1 sIgE increased by 24.8% from V1 to V7 (p = 0.001).

Consistent with these findings, the sIgE/sIgG4 ratio demonstrated a pronounced reduction after the first treatment phase, showing a 65.5% decrease when comparing V1 to V7 (p = 0.001) (Figure 5B).

Comparing immunogenicity results at V7 with the placebo group from the preceding trial at V7, Bet v1 sIgG4 increased 6.71‐fold and sIgE increased slightly (1.28‐fold). Also Bet v1 sIgG was 2.98‐fold higher after treatment with EP‐088_T502. Thus, the sIgE/sIgG4 ratio could be reduced by 78.2%.

### 3.5 Safety Results

Overall, no fatality and no serious AEs that were (possibly) related to the trial medication occurred during the trial. Due to the good safety profile that was demonstrated in the dose‐finding trial, no EpiPen was dispensed, and during the entire study period, epinephrine was not used. In total, 6 systemic allergic reactions (Grade I/II) were reported; all occurred in the first year of the study. As a consequence, the first injected doses were reduced to 0.1 mL and 0.2 mL in the subsequent year. Six SRs occurred in six patients (3.9%), with two of them being asthmatic (Table S1). In correlation with the total number of EP‐088_T502 injections (*N* = 343), SRs were observed in 0.35% of all injections.

Almost all immediate LRs were either 0 cm (30.7%) or of mild (< 5 cm) intensity (69.2%). Treatment with EP‐088_T502 did not cause any severe LRs (Table S2). Median wheal diameters ranged from 0 cm (V11, 12) to 0.75 cm (V3), with a maximum of 5.65 cm. In general, wheal diameters peaked at V3 but subsequently decreased at the following visits. In total, nine patients withdrew due to AEs, five of whom did so after experiencing SRs of Grade I‐II. Regarding late phase LRs, severe wheals accounted for less than 0.1% (*N* = 6) of all measurements (Grade III, wheal size > 20 cm); 0.2% (*N* = 16) of all wheals were moderate (Grade II, 10–20 cm; Table S3). In patients who received the intended treatment schedule (without dose split), mean wheal diameters were < 0.38 cm regarding late phase reactions. The most common unsolicited AEs, with a (possible) relationship to the treatment, were injection site reaction (24.5%) and injection site pruritus (23.9%).

## 4 Discussion

In this open follow‐up study, we investigated the efficacy and safety of EP‐088_T502 over 2 additional treatment years after the preceding dose‐finding trial. Thereafter the study concluded with 1 follow‐up year after discontinuation of treatment. Following the recommendations of Pfaar et al. [34], the CSMS during the peak birch pollen season was used to assess the clinical impact. In comparison to the 2020 baseline value placebo group of the T502‐SIT‐020 study, [24] treatment with EP‐088_T502 further reduced allergic symptoms and medication intake reflected by a reduction of the median CSMS, dSS, and dMS already after 1 year of treatment. After the second year of treatment, the CSMS decreased further. Upon discontinuation of treatment, at the peak birch pollen season in 2023, the CSMS remained low. That indicates the desired long‐term benefits of the SCIT. In two similar studies with EP‐088_T502, CSMS reductions of 33% (*p* = 0.002) [25] and 24.7% [24] compared to placebo could be shown within the first season after treatment. To point out the pronounced reduction of medication intake, in 2022, a median dMS of 0.04 was documented for patients from the original placebo group, demonstrating that the majority of patients did not use antiallergic medication in the second year of treatment. In a post hoc analysis considering the CSMS of patients who completed the entire 4 years of both studies, 2 years of treatment with EP‐088_T502 appeared to be efficacious, while a third year of treatment stabilised the level of improvement. During the follow‐up period without treatment, symptoms increased again, though to a low level clearly below the baseline year.

International guidelines [6, 29, 30] recommend 3 years of AIT in order to reach long‐term efficacy after treatment discontinuation. In 1999, Durham et al. [31] conducted a comparable study to this trial and concluded that 3 years of treatment are necessary for long‐term tolerance. After 3 years of treatment, reductions in symptom and medication scores compared to the control group could be shown and also persisted at 3 year follow‐up after discontinuation of treatment. In 2017, Scadding et al. [28] stated that 2 years of AIT are not sufficient to achieve sustained allergen tolerance in patients with ARC. Their study was also based on 2 treatment and 1 follow‐up year, SLIT and SCIT with native grass‐pollen extracts were compared with placebo. Scadding et al. compared the Total Nasal Symptom Score (TNSS) between AIT and placebo and in contrast to improvements during treatment, no significant differences between treatment groups at 1‐year follow‐up could be observed (SCIT vs. Placebo −17.8%, *p* = 0.10, SLIT vs. Placebo −5.6%, *p* = 0.62) [28].

Regarding QoL, the results show a consistent improvement from 2021 to 2023 compared to baseline values recorded in 2020.

Clinical efficacy was supported by analysing immunologic parameters, that are recognised as surrogate markers of a successful desensitisation [39]. The treatment with EP‐088_T502 increased production of *Bet v1* specific IgG4 (4.82‐fold compared to the baseline of the follow‐up study), which is also reflected in a reduction of the sIgE/sIgG4 ratio. Compared to the placebo group from the preceding trial, *Bet v1* specific IgG4 increased 6.71‐fold. This was accompanied by a reduction of the sIgE/IgG4 ratio (−78.2%). In their trial, Scadding et al. also observed immunologic changes that persisted in the follow‐up year as well, especially increased levels of sIgG4 [28], although the clinical impact could not be demonstrated.

The chemical modification of allergen extracts which leads to reduced allergenicity can be seen as an approach to enhance the safety of SCIT by reducing the occurrence of systemic reactions, which represent the most concerning risk of this type of therapy [16, 17]. The results of this trial show a good safety and tolerability with mainly mild local reactions and only few systemic reactions. The frequency of the latter (SR in 3.9% of the patients and 0.35% of the injections) is in line with large long‐term studies included in the EAACI Guidelines (0.1% of the injections and in 2.1% of the patients) [6] and the German S2k Guideline on AIT of 2022 (0.17% of the injections and 1.9%–2.9% of the patients) [40]. In the trial published by Scadding et al. where native pollen extracts were used, the numbers of documented SRs were higher (52.8% of SCIT patients, more injections). Compared to 2 SCIT trials using depigmented polymerised birch pollen extracts [41, 42], where systemic reactions occurred in more than 20% of the patients, the rate of these reactions in the current trial was comparatively low.

In the past decades, long treatment schedules associated with conventional AIT were necessary to reach clinical benefit. This can also be seen in the trials conducted by Durham and Scadding [28, 31], which required weekly to monthly injections over several years. As this leads to low adherence and high costs, there is still need for shorter schedules and reduced number of administrations [28, 43].

In this trial, 5 pre‐seasonal (short‐course) injections in each of the 2 treatment years achieved clinical efficacy paired with a good tolerability and safety profile. Considering the placebo group from the preceding trial [24] and comparing this group with all actively treated patients of this trial, we could show that 2 years of SCIT with EP‐088_T502 reduce allergic symptoms and medication intake. These findings suggest that 2 short courses of AIT at 10,000 mTU/mL in 2 consecutive years could be sufficient to induce sustained improvement in birch pollen allergic patients. Moreover, a short regimen aligns particularly well with the shorter pollen exposure period (4–6 weeks per year) experienced by patients allergic to birch pollen, compared to the longer exposure period (2–3 months) associated with grass pollen allergies. Further studies are necessary to assess whether the clinical benefits persist beyond 1 year and to determine the potential long‐term effects over an extended period (3–5 years). In addition, analyses from so‐called ‘real‐world’ observational trials may complement conclusions from these RCT [44, 45, 46].

The current trial was limited by several factors. Primarily, its open‐label design needs to be mentioned, but also interannual differences in pollen exposure, and centres and patients dropping out from this long‐term study may have played the role. According to considerations of the FDA [47] the protocol and statistical analysis plan were finalised prior to the start of the trial in order to protect integrity and reliability. As the study aimed to treat all patients with the most effective concentration of the preceding study, the open‐label design was inevitable. This design has inherent limitations in terms of a causal interpretation of treatment effects. Possible confounders that exist in temporal relation to the study duration, such as the variability of pollen exposure from year to year, but also changes in patient behaviour and the quality of medical care during the COVID‐19 period, may have had a causal influence not only on the severity of symptoms, but also, for example, on the use of rescue medication. Such factors naturally limit the informative value of the data collected. Mitigation procedures are described in Figure S1.

Another limitation regarding the interpretation of the results is the nature of statistical analyses, which is exploratory (not confirmatory) and includes post hoc analyses. The given *p*‐values, therefore, should be interpreted as exploratory and without alpha‐adjustment for multiple testing.

The number of participants decreased with the duration of the trial. This limits the generalisability of data since it was caused not only by centres deciding not to participate any longer after the first or second year of the trial, like in the case of one centre where the principal investigator passed away during the course of the trial. Also, patients may have based their decision to stop treatment on the personally perceived success of one pre‐seasonal course of injections, but also on insufficient relief from this short‐course treatment. The reasons are not documented. Both situations have an influence on the long‐term efficacy findings and may distort the interpretation of the results.

Against the existing assumption of Scadding and Durham and the recommendations in the guidelines, we found that 2 years of SCIT may be sufficient to induce sustained allergic response improvement in birch pollen allergic subjects. Reduced symptoms and medication need, also in the original placebo group, persisting into the follow‐up year, demonstrate the clinical benefit of treatment with EP‐088_T502. The majority of patients did not require anti‐allergic rescue medication throughout the birch pollen season following 2 years of AIT, but mild symptoms reappeared in the post‐treatment period, pointing towards a persistent treatment. Our findings might pave the way for improved and shorter treatment schedules which could increase adherence and reduce costs of AIT.

## Author Contributions

E.R., R.M., S.A., S.P., J.L.S., L.D., L.R. and M.C. have made substantial contributions to the conception and design of the clinical trial. E.R. and C.N. were responsible for the project management of the clinical trial and prepared the figures. F.H. wrote the manuscript. A.R. was involved in the writing, review, and editing. C.A. and H.S. were responsible for the acquisition and analysis of data. M.C. was the coordinating investigator, while L.K., O.P. and S.T. were principal investigators and made contributions to the conception and design of the clinical trial. All authors approved the final version of the manuscript before submission.

## Funding

This work was supported by Inmunotek S.L.

## Ethics Statement

The study upon which this analysis was based was approved by the leading ERC (Ethikkommission an der TU Dresden, Germany, reference number AMG ff‐EK‐416092020).

## Conflicts of Interest

R.M. reports grants and personal fees from Inmunotek during the conduct of the trial; personal fees from ALK, grants from ASIT biotech, personal fees from Allergopharma, personal fees from Allergy Therapeutics, grants and personal fees from Bencard, grants from Leti, grants, personal fees and non‐financial support from Lofarma, non‐financial support from Roxall, grants and personal fees from Stallergenes, grants from Optima, personal fees from Friulchem, personal fees from Hexal, personal fees from Servier, personal fees from Klosterfrau, non‐financial support from Atmos, personal fees from Bayer, non‐financial support from Bionorica, personal fees from FAES, personal fees from GSK, personal fees from MSD, personal fees from Johnson&Johnson, personal fees from Meda, personal fees and non‐financial support from Novartis, non‐financial support from Otonomy, personal fees from Stada, personal fees from UCB, non‐financial support from Ferrero, grants from Hulka, personal fees from Nuvo, grants and personal fees from Ursapharm, personal fees from Menarini, personal fees from Mundipharma, personal fees from Pohl‐Boskamp, grants from Cassella‐med GmbH & Co. KG, personal fees from Laboratoire de la Mer, personal fees from Sidroga, grants and personal fees from HAL BV, personal fees from Lek, personal fees from PRO‐AdWise, personal fees from Angelini Pharma, grants and non‐financial support from JGL, grants and personal fees from bitop, grants from Sanofi, personal fees from Menarini, outside the submitted work. M.C. declares honoraria for presentations from ALK‐Abelló, Allergopharma, AstraZeneca, Bencard Allergie/Allergy Therapeutics, GalaxoSmithKline, HAL Allergy, Leti Pharma, Novartis, Roxall, Sanofi‐Aventis, Stallergenes outside the submitted work. Other non‐financial interests: Member of German Society of Allergy (AeDA) and German Society of Oto‐Rhino‐Laryngology, Head and Neck Surgery DGHNO‐KHC. Coordinating investigator of the present clinical trial. L.K. reports grants and personal fees from Inmunotek during the conduct of the trial; grants and personal fees from Allergopharma, grants and personal fees from Viatris, personal fees from HAL Allergie, personal fees form ALK‐Abelló, grants and personal fees from LETI Pharma, grants and personal fees from Stallergenes, grants from Quintiles, grants and personal fees from Sanofi, grants from ASIT biotech, grants bromoform, personal fees from Allergy Therapeutics., grants from Astra‐Zeneca, grants and personal fees from GSK, grants from Inmunotek, personal fees from Cassella med, personal fees from Novartis, personal fees from Regeneron Pharmaceuticals, personal fees from ROXALL Medizin GmbH, outside the submitted work; and Membership: AeDA, DGHNO, Deutsche Akademie für Allergologie und klinische Immunologie, HNO‐BV, GPA, EAACI. O.P. reports grants for his institution during the conduct of the trial from Inmunotek S.L., Spain, and he reports grants and/or personal fees and/or travel support from AEDA, Alfried Krupp Krankenhaus, ALK‐Abelló, Allergopharma, Almirall, Altamira Therapeutics, ASIT Biotech, AstraZeneca, Bencard Allergie GmbH/Allergy Therapeutics, Blueprint, Breazy Health, Cliantha, Deutsche AllergieLiga e.V., Deutsche Forschungsgemeinschaft, Dustri‐Verlag, ECM Expro&Conference Management GmBH, Forum für Medizinische Fortbildung, Georg‐Thieme‐Verlag, GSK, HAL Allergy Holding B.V./HAL Allergie GmbH, Inmunotek, Ingress Health, Institut für Disease Management, IQVIA Commercial, Japanese Society of Allergology, Königlich Dänisches Generalkonsulat, Laboratorios LETI/LETI Pharma, Lilly, Lofarma, Medizinische Hochschule Hannover, med update europe GmbH, Meinhardt Congress GmbH, Novartis, Paul‐Ehrlich‐Institut, Paul‐Martini‐Stiftung, PneumoLive, Pohl‐Boskamp, Procter & Gamble, Red Maple Trials Inc., Regeneron, RG Aerztefortbildung, ROXALL Medizin, Sanofi Aventis, Sanofi Genzyme, Springer Publisher, Stallergenes Greer, streamedup! GmbH, Technical University Dresden, John Wiley & sons publishers, Wort & Bild Verlag, Verlag ME; all outside the submitted work, Oliver Pfaar is Vice President of the European Academy of Allergy and Clinical Immunology (EAACI), a member of EAACI Excom as well as a member of the external board of directors of the German Society of Allergy and Clinical Immunology (DGAKI); coordinator, main‐ or co‐author of different position papers and guidelines in rhinology, allergology and allergen‐immunotherapy; and he is Editor‐in‐Chief of Clinical Translational Allergy and Associate Editor of Allergy. S.Z. reports grants and personal fees from Inmunotek during the conduct of the trial; grants from Palas GmbH, grants and personal fees from Allergy Therapeutics GmbH, grants and personal fees from Böhringer Ingelheim, personal fees from Novartis GmbH, personal fees from Lofarma GmbH, personal fees from IMS HEALTH GmbH & Co. OHG, personal fees from GSK, personal fees from Stallergenes, personal fees from Engelhard Arzneimittel, personal fees from Sanofi‐Pasteur, personal fees from AstraZeneca, personal fees from Erydel, outside the submitted work. S.P. is an employee of Inmunotek, J.L.S. and M.C. are shareholders of Inmunotek. All authors had full access to all the data in this trial and take complete responsibility for the integrity of the data and accuracy of the data analysis.

## Supporting information


**Data S1:** Inclusion and non‐inclusion criteria of the T502‐SIT‐041 trial.
**Table S1:** Listing of systemic reactions (SR) occurring during the T502‐SIT‐041 trial. RM: rescue medication (Fexofenadine, 180 mg).
**Table S2:** Listing of immediate local reactions (LR) occurring ≤ 30 min following injections of EP‐088_T502: mean wheal diameters [cm] averaged over all treatment visits. N: number of measurements.
**Table S3:** Listing of late phase local reactions (LR) occurring > 30 min following injections of EP‐088_T502: mean wheal diameters [cm] averaged over all treatment visits. N: number of measurements.
**Table S4:** CSMS values of patients who could be analysed continuously from 2020 to 2023.
**Table S5:** CSMS values during the peak birch pollen seasons 2020–2023. Post hoc analysis of 20 patients from the placebo group in the previous study, who completed the study in 2023 alongside pooled study groups from 2021 to 2023, who also completed the study in 2023.
**Figure S1:** Timing of SARS‐CoV‐2 and/or flu vaccinations during the treatment phase.
**Figure S2:** CSMS during the peak birch pollen seasons 2020–2023. Post hoc analysis of 20 patients from the placebo group in the previous study, who completed the study in 2023 alongside pooled study groups from 2021 to 2023, who also completed the study in 2023. Data is presented as box plots including minimum, maximum, median, 25th and 75th percentile. *Mann Whitney U tests were used for pairwise comparisons providing the unadjusted *p* values for each comparison. **Kruskal Wallis test was employed to identify differences across multiple comparisons indicating *p* value.
**Figure S3:** Mean RQLQ scores at V7 (before the birch pollen season 2021), V8 (at the peak of the birch pollen season 2021), V15 (before the birch pollen season 2022), V16 (at the peak of the birch pollen season 2022), V18 (before the birch pollen season 2023) and V19 (at the peak of the birch pollen season 2023). Data is presented as box plots including minimum, maximum, median, 25th and 75th percentile. RQLQ, rhinoconjunctivitis quality‐of‐life questionnaire.

## Data Availability

The data that support the findings of this study are available from the corresponding author upon reasonable request.
