# Supplementary material for: Sustained Clinical Improvement in Birch Pollen Allergy After Two Pre‐Seasonal Short Courses of Allergen‐Specific Immunotherapy: A Long‐Term Open‐Label Extension Study
Source: Clin Exp Allergy. 2026 Apr 30;56(8):893–903. doi: 10.1111/cea.70323 (PMC13429321; doi:10.1111/cea.70323)
Supplement: Supplementary file 1 — Data S1: Inclusion and non‐inclusion criteria of the T502‐SIT‐041 trial. Table S1: Listing of systemic reactions (SR) occurring during the T502‐SIT‐041 trial. RM: rescue medication (Fexofenadine, 180 mg). Table S2: Listing of immediate local reactions (LR) occurring ≤ 30 min following injections of EP‐088_T502: mean wheal diameters [cm] averaged over all treatment visits. N: number of measurements. Table S3: Listing of late phase local reactions (LR) occurring > 30 min following injections of EP‐088_T502: mean wheal diameters [cm] averaged over all treatment visits. N: number of measurements. Table S4: CSMS values of patients who could be analysed continuously from 2020 to 2023. Table S5: CSMS values during the peak birch pollen seasons 2020–2023. Post hoc analysis of 20 patients from the placebo group in the previous study, who completed the study in 2023 alongside pooled study groups from 2021 to 2023, who also completed the study in 2023. Figure S1: Timing of SARS‐CoV‐2 and/or flu vaccinations during the treatment phase. Figure S2: CSMS during the peak birch pollen seasons 2020–2023. Post hoc analysis of 20 patients from the placebo group in the previous study, who completed the study in 2023 alongside pooled study groups from 2021 to 2023, who also completed the study in 2023. Data is presented as box plots including minimum, maximum, median, 25th and 75th percentile. *Mann Whitney U tests were used for pairwise comparisons providing the unadjusted p values for each comparison. **Kruskal Wallis test was employed to identify differences across multiple comparisons indicating p value. Figure S3: Mean RQLQ scores at V7 (before the birch pollen season 2021), V8 (at the peak of the birch pollen season 2021), V15 (before the birch pollen season 2022), V16 (at the peak of the birch pollen season 2022), V18 (before the birch pollen season 2023) and V19 (at the peak of the birch pollen season 2023). Data is presented as box plots including minimum, maximum, median, 25th [file CEA-56-893-s001.docx]

Supporting Information

**Data S1:** Inclusion and non-inclusion criteria of the T502-SIT-041 trial

**Inclusion Criteria**

Patients must meet all of the following inclusion criteria in order to participate in this study:

- Signed and dated Informed Consent Form by a legally competent patient,
- Participation in the T502-SIT-020 trial,
- Being in good physical and mental health,
- Confirmed normal renal and liver function, including non-clinically significant deviations outside the reference ranges (< grade 2 according to the Food and Drug Administration (FDA) Guidance for Industry for preventive Vaccine Trials (FDA 2007) at screening visit*),
- For females: non-pregnant, non-lactating with adequate contraception, or females unable to bear children (i.e. tubal ligation, hysterectomy, or post-menopausal (defined as a minimum of one year since the last menstrual period)),
- For asthmatic patients: confirmed diagnosis of controlled asthma during the treatment period according to Global Initiative for Asthma (GINA) guidelines (steps 1-3, GINA 2014) Forced Expiratory Volume in 1 second (FEV1) ≥ 80% of the patient’s reference value or Peak Expiratory Flow (PEF) ≥ 80% of the patients’ individual optimal value (for asthmatic patients only).

**Non-Inclusion criteria**

Patients meeting the following non-inclusion criteria are not allowed to participate in the study

or have to be excluded during the course of the study:

- Simultaneous participation in other clinical trials or previous participation within 30 days before inclusion,
- No participation in the T502-SIT-020 study,
- Ongoing immunotherapy with birch pollen allergens or any other allergens,
- Being in any relationship or dependence with the Sponsor, Contract Research Organisation (CRO) and/or Investigator,
- Inability to understand instructions/study documents,
- Patients who do not have access to a smartphone/tablet (iOS or Android),
- History of severe systemic reactions and/or anaphylaxis, including to food (e.g. peanut, marine animals) or to Hymenoptera venom (e.g. bee, wasp stings) or to medication (e.g. penicillin), etc.,
- History of hypersensitivity to the excipients of the investigational product or placebo,
- Mild persistent to severe persistent asthma, partly controlled or uncontrolled asthma according to GINA guidelines (GINA 2014) during the treatment period,
- Chronic asthma or emphysema, particularly with a forced expiratory volume in 1 second (FEV1) < 80% of the patient’s reference value (European Community for Steal and Cole [ECSC]) or Peak Expiratory Flow (PEF) < 80% of the patients’ individual optimal value,
- History of a respiratory tract infection and/or exacerbation of asthma within 4 weeks before the screening visit V1,
- Patients who have suspicion or symptoms of a Severe acute respiratory syndrome coronavirus type 2 (SARS-COV-2) infection, who have had contact with a confirmed case of Coronavirus disease (COVID-19), or returned from a COVID-19 risk region in the 2 weeks prior to the screening visit V1,
- Patients with acute allergic rhinoconjunctivitis due to other environmental allergens during the study period,
- History of significant renal disease or chronic hepatic disease,
- Malignant active disease (ongoing or within the five past years),
- Severe autoimmune disease, Immune defects including immunosuppression, immunopathies or vaccination during the entire treatment periods December 2020 to March 2021 and December 2021 to
- March 2022, except influenza and SARS-CoV-2 vaccinations – see Chapter 9.4.7.1 ‘Non-allowed medication and procedures’,
- Use of systemic immunosuppressive medications (e.g., methotrexate or cyclosporine A) or blood transfusion one month before screening,
- General inflammatory, severe acute or chronic inflammatory diseases,
- Other chronic diseases such as severe congestive heart failure, cardiovascular insufficiency, active gastric ulcer, inflammatory bowel disease, uncontrolled diabetes mellitus, etc.,
- Intake of antidepressant drugs with potent antihistamine properties such as tricyclic antidepressants (e.g. doxepin, amitriptyline, desipramine, imipramine, etc.),
- Administration or planned administration of anti-IgE antibodies, mast cell stabilisers or anti-leukotriene agents,
- Intake of beta-blockers/ACE inhibitor medication (angiotensin-converting enzyme inhibitor),
- Active tuberculosis,
- Having any contraindication for the use of adrenaline (including hyperthyroidism),
- Known positive serology to Human Immunodeficiency Virus-1/2, Hepatitis B Virus or Hepatitis C Virus,
- Females who are pregnant, lactating, or of child-bearing potential and not using an adequate contraceptive method which is defined in chapter 9.3.1 ‘Inclusion Criteria’,
- Administration of corticosteroids (oral, topic or nasal) or of anti-histaminic drugs within the time period preceding the trial (screening visit), as defined in the protocol, exception made for routine (previously prescribed) control medication for asthmatic patients,
- Clinically relevant laboratory values, i.e. grade ≥ 2 according to the FDA Guidance for Industry for preventive Vaccine Trials (FDA 2007) at screening visit,
- Patients that the Investigator believed wouldn’t have complied with the study protocol (patients with known alcohol or drug abuse or with a history of a serious psychiatric disorder as well as patients unwilling to give informed consent or to abide by the requirements of the protocol).

**COVID-19 risk mitigation:**

Due to the Covid-19 pandemic that occurred during the conduct of the study in Germany, vaccinations against SARS-CoV-2 and influenza were – in contrast to other vaccinations – allowed during the treatment phase. If these vaccinations were given during the treatment phase, the following intervals had to be maintained:


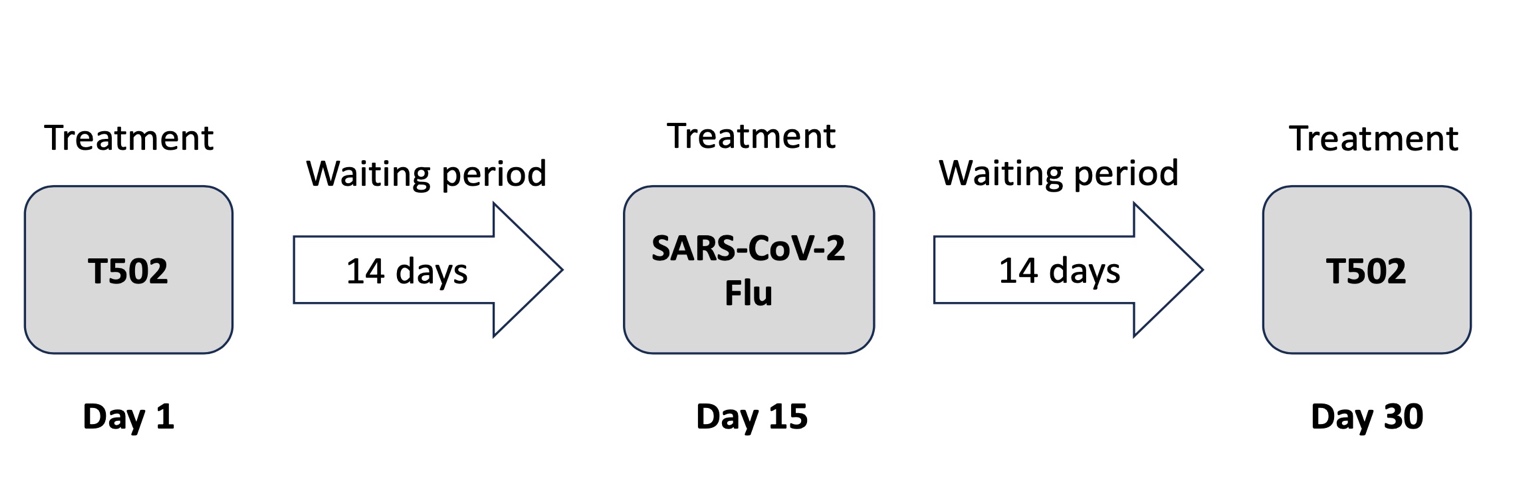


Figure S1: Timing of SARS-CoV-2 and/or flu vaccinations during the treatment phase.

**Supporting Information B:** safety results

Table S1: Listing of systemic reactions (SR) occurring during the T502-SIT-041 trial. RM: rescue medication (Fexofenadine, 180 mg)

| **Treatment group in T502-SIT-020 trial** | **Description of SR** | **Severity** | **Outcome** | **Continuation / discontinuation** |
| --- | --- | --- | --- | --- |
| 1000 mTU/mL | late phase SR Grade I  after V2:  (LLT) urticarial rash and itchy rash | moderate | resolved within 5 hours after treatment with intravenous electrolytes and Fenistil | discontinued |
| Placebo | late phase SR Grade I  after V2:  generalized itching and cervix erythema (LLT) | moderate | resolved within half a day without treatment | discontinued |
| 10 000 mTU/mL | late phase SR Grade I following V2: rash on trunk and redness (LLT) | moderate | resolved within 4 hours after intake of RM | discontinued |
| 1000 mTU/mL | immediate SR Grade II with asthma after V3 | mild | resolved after 20 minutes after treatment with Salbutamol | continued |
| Placebo | immediate SR Grade II after V2 with shortness of breath, cough and asthma attack (LLT) | severe | resolved within 2 hours after treatment with RM and intravenous administration of Tavegil and Solu-Decortin | discontinued |
| Placebo | immediate SR Grade II during V3 with cough, eye pressure, conjunctivitis, facial flushing | severe | resolved within 3 hours after inhalation of Salbutamol and Pulmicort (Budesonide), oral administration of Xusal (Levocetirizine Dihydrochloride) and intravenous administration of Fenistil and prednisolon | discontinued |

Table S2: Listing of immediate local reactions (LR) occurring ≤30 minutes following injections of EP-088_T502: mean wheal diameters [cm] averaged over all treatment visits. N: number of measurements

| **Wheal size [cm]** | **EP-088_T502** | |
| --- | --- | --- |
|  | **N** | **%** |
| 0 cm | 526 | 30.7 |
| <5 cm (mild) | 1185 | 69.2 |
| 5-10 cm (moderate) | 2 | 0.1 |
| >10 cm (severe) | 0 | 0 |
| Total | 1713 | 100 |

Table S3: Listing of late phase local reactions (LR) occurring >30 minutes following injections of EP-088_T502: mean wheal diameters [cm] averaged over all treatment visits. N: number of measurements

| **Wheal size [cm]** | **EP-088_T502** | |
| --- | --- | --- |
|  | **N** | **%** |
| 0 cm | 6145 | 88.5 |
| <10 cm (mild) | 776 | 11.2 |
| 10-20 cm (moderate) | 16 | 0.2 |
| >20 cm (severe) | 6 | 0.1 |
| Total | 6943 | 100 |

**Supporting Information C:** Post-hoc analysis

Table S4: CSMS values of patients who could be analysed continuously from 2020 to 2023.

|  | | | CSMS (Peak 2020) | CSMS (Peak 2021) | CSMS  (Peak 2022) | CSMS (Peak 2023) |
| --- | --- | --- | --- | --- | --- | --- |
| Treatment group in T502-SIT-020 study | Placebo n=20 | Median | 1.48 | 0.74 | 0.43 | 0.75 |
|  |  | Mean + SD | 1.44+0.97 | 0.90+0.82 | 0.71+0.84 | 0.88+0.65 |
|  | 1000 n=27 | Median | 1.19 | 0.64 | 0.73 | 0.90 |
|  |  | Mean + SD | 1.23+0.63 | 0.81+0.68 | 0.77+0.50 | 0.91+0.70 |
|  | 3000  n=25 | Median | 1.09 | 0.67 | 0.62 | 0.96 |
|  |  | Mean + SD | 1.33+0.93 | 0.73+0.53 | 0.76+0.66 | 1.04+0.75 |
|  | 10 000  n=22 | Median | 0.77 | 0.47 | 0.80 | 0.79 |
|  |  | Mean + SD | 1.02+0.90 | 0.74+0.89 | 0.87+0.68 | 0.98+0.68 |

Table S5: CSMS values during the peak birch pollen seasons 2020-2023. Post-hoc analysis of 20 patients from the placebo group in the previous study, who completed the study in 2023 alongside pooled study groups from 2021-2023, who also completed the study in 2023

|  | | Placebo T502-SIT-020 | Pooled study  groups  T502-SIT-041 | | |
| --- | --- | --- | --- | --- | --- |
|  |  | 2020 | 2021 | 2022 | 2023 |
| CSMS_Peak | Total N | 20 | 94 | 94 | 94 |
|  | Valid N | 20 | 94 | 94 | 94 |
|  | Mean | 1.44 | 0.79 | 0.78 | 0.95 |
|  | Standard Deviation | 0.97 | 0.72 | 0.66 | 0.69 |
|  | Standard Error of Mean | 0.22 | 0.07 | 0.07 | 0.07 |
|  | 95.0% Lower CL for Mean | 0.98 | 0.64 | 0.64 | 0.81 |
|  | 95.0% Upper CL for Mean | 1.89 | 0.94 | 0.91 | 1.10 |
|  | Minimum | 0.03 | 0.00 | 0.00 | 0.00 |
|  | Median | 1.48 | 0.59 | 0.63 | 0.87 |
|  | Maximum | 3.57 | 3.71 | 2.76 | 3.17 |
|  | Percentile 25 | 0.75 | 0.22 | 0.23 | 0.42 |
|  | Percentile 75 | 1.84 | 1.03 | 1.17 | 1.40 |
| p Overall comparison with Kruskal Wallis | | | |  | 0.004 |
| p Pairwise comparisons with Mann Whitney U test | | | | 2020 vs 2021 | 0.003 |
|  |  |  |  | 2020 vs 2022 | 0.003 |
|  |  |  |  | 2020 vs 2023 | 0.025 |
|  |  |  |  | 2021 vs 2022 | 0.953 |
|  |  |  |  | 2021 vs 2023 | 0.036 |
|  |  |  |  | 2022 vs 2023 | 0.061 |


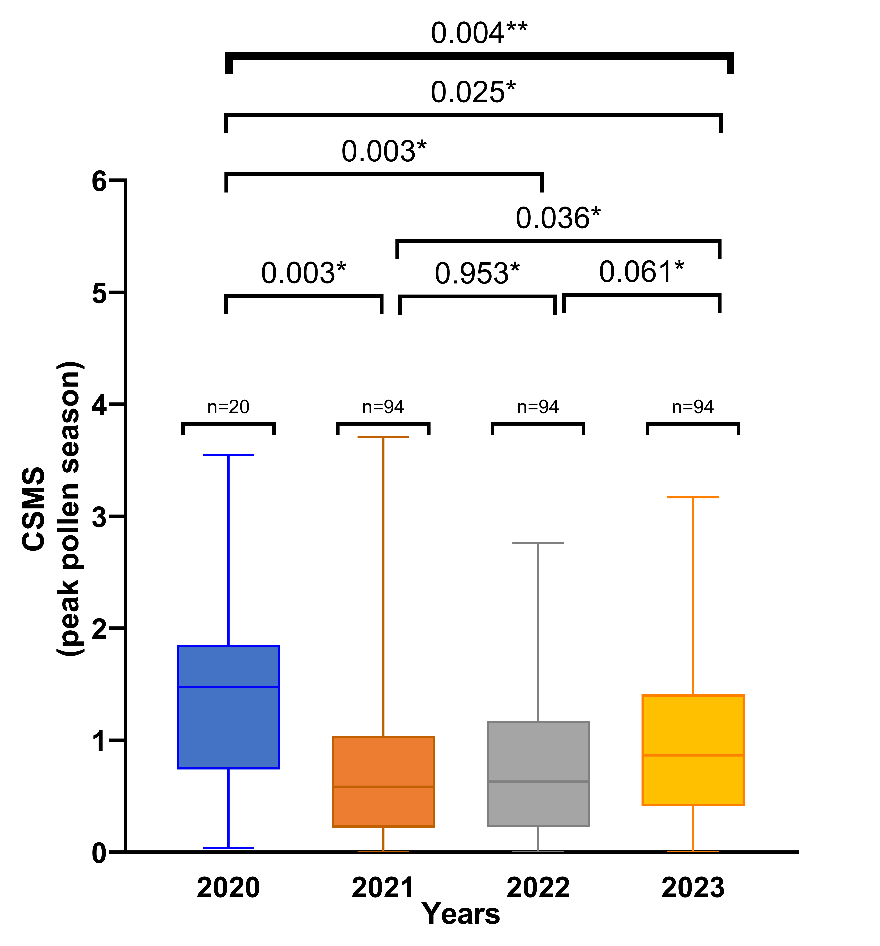


Figure S2: CSMS during the peak birch pollen seasons 2020-2023. Post-hoc analysis of 20 patients from the placebo group in the previous study, who completed the study in 2023 alongside pooled study groups from 2021-2023, who also completed the study in 2023. Data is presented as box plots including minimum, maximum, median, 25^th^ and 75^th^ percentile. *Mann Whitney U tests were used for pairwise comparisons providing the unadjusted p values for each comparison. **Kruskal Wallis test was employed to identify differences across multiple comparisons indicating p value

**Supporting Information D:** Health-related Quality of Life (QoL)


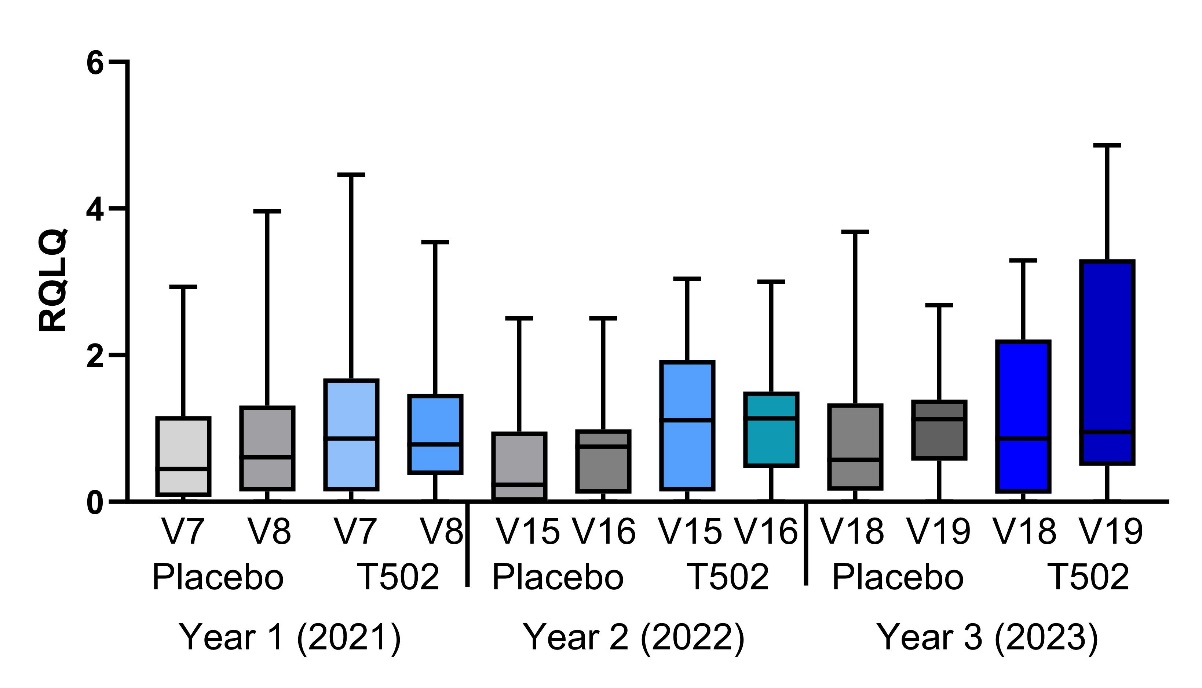


Figure S3: Mean RQLQ scores at V7 (before the birch pollen season 2021), V8 (at the peak of the birch pollen season 2021), V15 (before the birch pollen season 2022), V16 (at the peak of the birch pollen season 2022), V18 (before the birch pollen season 2023), and V19 (at the peak of the birch pollen season 2023). Data is presented as box plots including minimum, maximum, median, 25^th^ and 75^th^ percentile. RQLQ, rhinoconjunctivitis quality of life questionnaire.

**List of additional investigators of the BetMan study group:**

Elke Decot, Thomas Ginko, Gerhard Hoheisel, Jürgen Palm, Rainer Reiber, Udo Schäfer, Uta Thieme, Jörg Winkler, Yuri Yarin, Christian Schlenska, Daniela Kasche
